# Compositional and Functional Characteristics of Swine Slurry Microbes through 16S rRNA Metagenomic Sequencing Approach

**DOI:** 10.3390/ani10081372

**Published:** 2020-08-07

**Authors:** Himansu Kumar, Yu Na Jang, Kwangmin Kim, Junhyung Park, Min Woong Jung, Jong-Eun Park

**Affiliations:** 1 Division of Animal Genomics and Bioinformatics, National Institute of Animal Science, RDA, Wanju 55365, Korea; himanshu.genetics@gmail.com; 2 Animal Environment Division, National Institute of Animal Science, RDA, Wanju 55365, Korea; jyn0316@korea.kr; 3 3 BIGS CO. LTD., Hwaseong 18454, Korea; kmkim@3bigs.com (K.K.); jhpark@3bigs.com (J.P.)

**Keywords:** microbiota, 16S rRNA sequencing, Greengenes database, QIIME

## Abstract

**Simple Summary:**

The goal of present study was to evaluate and characterize the microbes present in the pig slurry. Samples were collected from three different slurry pits of a pig farm at different storing time points. With the help of a 16S rRNA metagenomic sequencing approach, a detailed catalogue of bacterial composition was reported. The biochemical pathways were investigated to explore the functional patterns of microbes. This study may help to understand the changes in microbial diversity with the storage time of pig slurry.

**Abstract:**

Traditionally slurry is used as source of nitrogen, phosphorous, and potassium in bio fertilizers to improve crop production. However, poorly managed slurry causes a hazardous effect to the environment by producing greenhouse gases, causing the eutrophication of water bodies, and polluting the groundwater. It has been largely reported that the microbial presence in slurry causing a diverse effect on its storage and disposal system. However, the diversity of bacterial populations in pig slurries remains largely unexplored. Here we report the bacterial diversity present in the slurry from slurry pits, and the effect of storage time on bacterial population. We collected 42 samples from three different pig slurry pits, as three replicates from each one until the 14th week. We used the 16S rRNA, Quantitative Insights Into Microbial Ecology (QIIME) and Phylogenetic Investigation of Communities by Reconstruction of Unobserved States (PICRUSt) protocols for the metagenomic downstream analysis. Taxonomic annotation using the Greengenes metagenomic database indicated that on an average 76.2% *Firmicutes*, 14.4% *Bacteroidetes*, 4.9% *Proteobacteria,* etc. microbial populations were present. Comparative microbial analysis showed that the population of *Firmicutes* decreased from the first to the 14th week, whereas the population of *Bacteroidetes* increased from the first to the 14th week. Through principal coordinate analysis (PCoA), (linear discriminant analysis effect size (LEfSe), and Pearson’s correlation analysis, we found microbial biomarkers according to the storage time point. All bacterial populations were well clustered according to the early, middle, and last weeks of storage. LEfSe showed that *Actinobacteria*, *Lachnospiraceae*, *Ruminococcaceae*, and *Bacteroidia* are dominantly present in first, seventh, ninth, and 14th week, respectively. *Lachnospiraceae* and *Ruminococcaceae* are ubiquitous gastrointestinal non-pathogenic bacteria. KEGG pathways, such as membrane transport, carbohydrate and amino acid metabolism, genetic replication and repair, were significant among all samples. Such a KEGG pathway may indicate the association between the host organism’s metabolic activity and the microbes present in the gastro intestinal tract (GIT).

## 1. Introduction

Most of the world depends upon pig to fulfill their animal protein demand as reported by the Food and Agricultural Organization (FAO) of the United Nations [1]. It was reported in 2011 that South Korea ranked in fifth position in the world in terms of pork consumption. Pig farming is considered as a significant source of income in Korean rural areas [2]. The agriculture department of South Korea reported in 2014 that about 16 million pigs were slaughtered in 2013 to meet the demand of pork. However, a major setback in pork industry had been observed due to outbreak of foot and mouth disease in 2010–2011. The principal source of infections for pigs is the contact with excretory materials from infected animals. The cleanliness and hygiene of pig farming can be achieved by an efficient and strict slurry management system. The huge production of swine slurry and their management is an utmost need to understand the pig farming requirements [3]. Stored slurry releases the severe offensive odor into the environment because it comprises of microbes, fatty acids, indoles, phenols, ammonia and sulphur-containing compounds [4,5]. Poorly managed slurry pits can cause the hazardous effects to the environment by producing greenhouse gasses, the eutrophication of water reservoirs, and depositing pollutants into water bodies. The bacteria present in slurry break it into multiple gases such as carbon monoxide, methane, ammonia, and hydrogen sulfide. These gases represent a risk to human and animal health by asphyxiation, nausea, unconsciousness, etc. Major challenges for swine slurry waste management include controlling the dispersion of pathogenic microorganisms [6,7,8,9].

The most traditionally and commonly used method for the recycling of swine slurry is to spread it on agricultural farming land to improve the crop production [10,11]. It has been reported that slurry is a rich source of nitrogen, phosphorous, and potassium. The microbes present in slurry play a very significant role in the pig’s life [8]. It is estimated that 35–70% of herds and 4.5–100% of individual swine carry pathogenic microbes [12,13,14]. Pathogenic microbial contamination through pig slurry to water and food represent a serious threat to human health as well as other animal’s health [15,16]. Slurry samples of pig also have the potential to provide information about pig intestinal parasites, genetics, and diet.

By considering the wide impact of slurry on the environment, in this study, we used metagenomic approaches to investigate the slurry microbial composition. We conducted time series sampling and DNA extraction starting from one to 14 weeks and used a 16S rRNA high-throughput metagenomic sequencing approach. We considered the duration of sampling till the 14th week by considering 2–3 months of general storage practice of slurry by the farmers. The storage of slurry causes the growth of microbes which are involved in anaerobic degradation and methane production. There are limited studies available about the association between microbe composition and functional patterns. Therefore, understanding the effect of microbes in slurry storage is needed to mitigate the strategies of slurry management.

Through the bioinformatics analysis of metagenomic reads, we elucidated their microbial taxonomic structures through 16S rRNA metagenomic sequencing approach. The 16S rRNA sequencing approach is high-throughput sequencing and is widely used to predict the phylogenetic and taxonomic diversity in various samples such as intestinal, host-associated, fecal, water bodies, slurry, etc. The generally shotgun metagenomic approach has been used to estimate the functional annotations of microbes. However, in this study, we used 16S rRNA for the overall functional inference of microbes present in the pig slurry. Many tools such as Tax4Fub, Piphillin, Faprotax, Paprica, and PICRUSt are freely available for taxonomy annotations. The accuracy of such inferences or predictions is largely dependent upon the genomic source available in public databases. We also identified metagenomic biomarkers through LEfSe (linear discriminant analysis effect size) and co-expression analysis, which indicate the significant abundance among sample groups.

## 2. Materials and Methods

### 2.1. Sample Preparation, Demultiplexing and Quality Filtering

All experiments were carried out according to the standard protocol approved by the NIAS animal care approval committee with the approval number 2018–262. We considered a total of 98 pigs of 77 days-old of 25 ± 2.50 kg in weight, and provided the same diet to each pig. The pigs were reared in seven different pens, and their slurry into was stored into three different pits, uniformly collected from seven pens at the National Institute of Animal Science (NIAS), South Korea. The temperature of the slurry pits varied from 28 °C to 29 °C, and the humidity varied from 81 to 89%. One sample from each slurry pit was collected at every week till the 14th week (14× = 42) and stored at −80 °C till the DNA extraction experiment was conducted [17]. The pH and moisture content of the slurry were measured during every sample collection time point. The DNA was extracted from each week’s sample through the Fast DNA Spin Kit (MP Bio, USA), by following the manufacturer’s standard protocol and we stored the DNA at −20 °C till further use. For the 16S rRNA metagenomic sequencing, the V4 region of 16S rRNA gene was amplified from the extracted DNA with the help of universal primers (5′-GGACTACHVGGGTWTCTAAT-3′ and 5′-GTGCCAGCMGCCGCGGTAA-3′). Unique barcode sequences were inserted between the adapter and linker for the simultaneous sequencing of multiple samples. Paired-end reads were preprocessed by removing the barcode sequences, linker, and PCR primers. Further reads were quality trimmed and demultiplexed by keeping Phred Score > 20 [18,19].

### 2.2. Operational Taxonomic Unit (OTU) Clustering and Taxonomy Assignment

The processed reads were analyzed by the QIIME tool [20]. The reads were aligned with the databases Greengene v13.8 for assigning the OTU (operational taxonomic unit) [21]. Open-reference OTU cluster analysis with USEARCH (v7.0.1090) and PyNAST was used for alignment by keeping 97% sequence identity parameter to pick the OTU [22,23]. Relatively less abundant (<0.05%) and rare OTUs were removed to normalized the OTU table and used for downstream analysis through QIIME. Each OTU of the 16S rRNA sequence was assigned a taxonomy ID on the basis of alignment with the reference database and NCBI by using the RDP classifier program (v2.2) [24,25]. Bar chart and phylogenetic trees were generated from the OTU identifier table [26]. Comparative chart for 1–14 weeks were generated according to phylum, class, order, family, genus, and species level [27].

### 2.3. Microbial Diversity Analysis

The observed OTUs were used for diversity analysis through alpha and beta diversity [28]. The alpha diversity metrics was computed by using a rarefied OTU table. Rarefaction curves of varying sample size were generated by using Observed_otus, Chao1, ace, Simpson, Shannon, and Goods_Coverage [29]. The ratio between the regional and local microbial population was estimated by the Unifrac distance for beta diversity analysis through the QIIME protocol [30]. Principal coordinate analysis (PCoA) plots were also generated by performing jackknifed unweighted pair group method arithmetic mean (UPGMA) clustering through Bray–Curtis distance matrices [31,32].

### 2.4. Feature Selection through Linear Discriminant Analysis (LDA) Effect Size (LEfSe)

Metagenomic features, such as class, organisms, OTU, genes, and functions can be predicted through LDA–LEfSe methods. We tried to determine the biomarker microbes among 14 groups using relative abundances. We kept the criteria of the LDA score for discriminative features >3.0, and the alpha value for the factorial Kruskal–Willis test among the classes was <0.05.

### 2.5. Investigation of Co-Occurring and Mutually Exclusive Microbes

We investigated the associations of individual bacteria in terms of degree of interaction. For making the network visualization simple, we selected limited weeks of the sample (1, 4, 7, 10 and 14 week). A *p*-value was calculated from the OTU count distribution, and the node group week wise relationships with *p*-values more than 0.05 were removed. We only kept the fractions of total relationships which were more significant than those expected by chance.

### 2.6. Microbial Functional Profiling

We investigated the relative abundance of functional categories based on the assigned taxa of the OTU [33]. PICRUSt was designed to interpret the 16S amplicon sequencing data in terms of biological pathways. PICRUSt predicts the abundance of gene categories (COGs) and metabolic pathways (KEGG). Identified function of genes was investigated and visualized with STAMP software for relative abundance [34].

### 2.7. Data Availability

The sequencing data of the swine slurry samples have been submitted in the NCBI Sequencing Read Archive (SRA) under the bio-project PRJNA577738.

## 3. Results

### 3.1. Sample Information and Read Statistics

**Figure 1:**
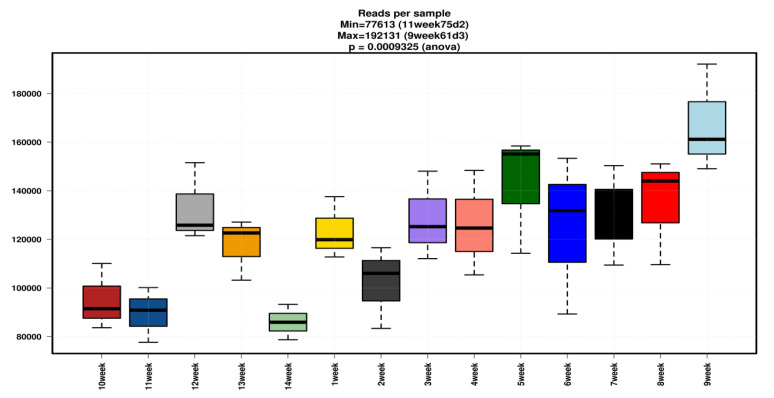
Boxplot of week wise sample showing the reads per sample of each group, X axis indicates the number of weeks and the Y axis indicates the number of reads. Different colors are just representing the differentiation among samples.

The total number of samples for 16S rRNA sequencing was 42 (*n* = 42). Read counts for all sequences were varied from 157,447 (sixth week) to 259,960 (ninth week), GC (%) 52 to 53, Q20 (%) 97 to 98, and Q30 (%) ranges to 91–93. Detail values of total bases, read count, N (%), Q20 (%), Q30 (%) for all individual samples are shown in supplementary Table S1. The box plot for reads per sample is shown in Figure 1.

**Figure 2:**
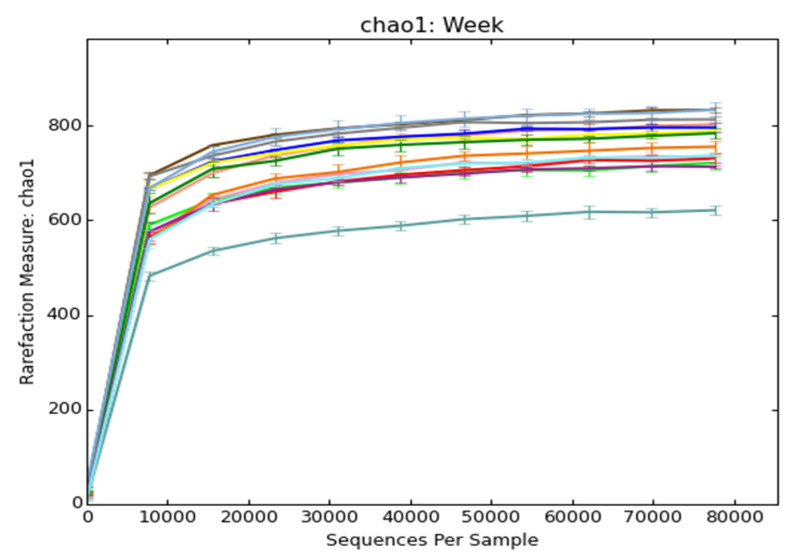
Rarefaction curve through chao1 for each week sample pooled from the three slurry pits; overall, the plot of all weeks shows a trend of consistent richness. The ending of the curves indicates the number of sequences obtained, as shown on the X axis. Rarefaction curve through the Observed_species, Shannon, and Simpson indexes are shown in the Supplementary Figures.

Quality-checked and filtered reads were considered for the OTU assignment, we found 485–825 OTUs from all samples. The lowest (485) OTUs were from the ninth week samples, whereas the highest (825) were from 11th week. A total of 9858 OTUs were assigned from the sequences of all samples. Details about the number of OTUs, Chao1, Shannon, Simpson, and Goods Coverage are shown in Supplementary Table S2. The rarefaction curve showed that the data of all the samples’ approaches to completeness in the curve plateaus, as shown in the plot of Chao1 (Figure 2), Observed_species, Shannon, and Simpson curves are shown in the Supplementary Figure S3.

### 3.2. Multivariate Analysis

**Figure 3:**
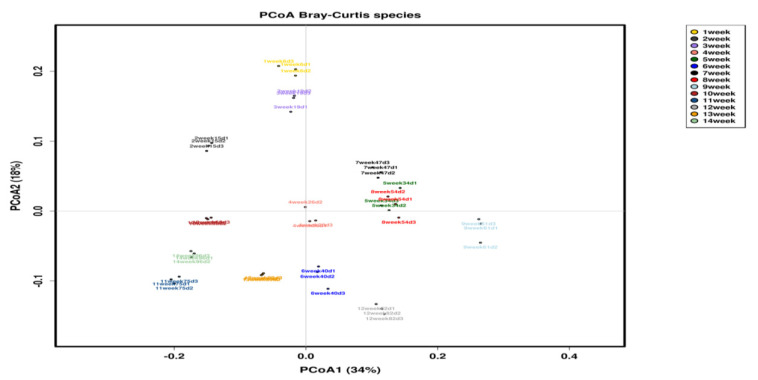
Representation of the beta-diversity through the Weighted UniFrac principal coordinate analysis (PCoA), plot and the different colors indicate the different week of sample.

Multivariate analysis of beta-diversity for community structure and diversity through principal coordinate analysis (PCoA), we observed a clear separation among the multiple weeks of slurry samples, as shown in Figure 3. The Bray–Curtis analysis of the OTU shows almost all the samples clustered according to their sample group.

### 3.3. Microbial Taxonomy Annotation

**Figure 4:**
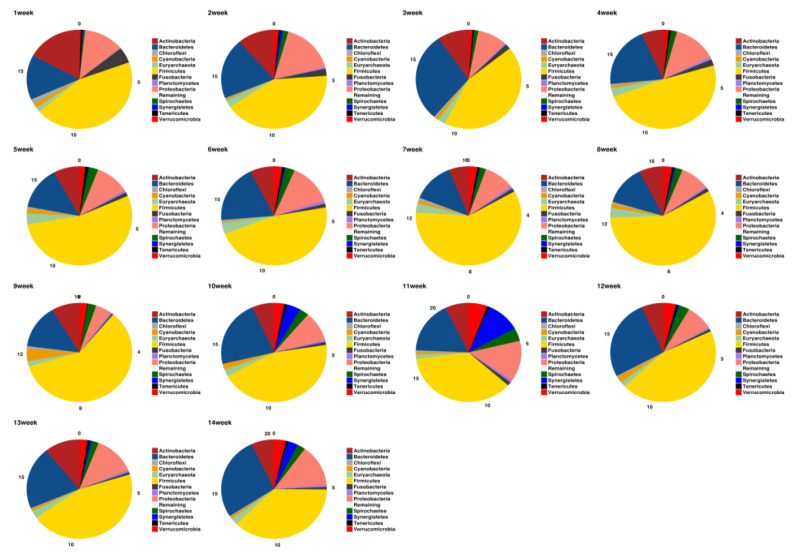
Taxonomic annotation of the polled sample from the three slurry pits, the abundance of *Firmicutes*, *Bacteroidetes*, etc., can be observed throughout the storage duration (first to 14th week). Each color represents the relative abundance (%) for each phylum.

Taxonomy composition of each sample from the phylum to species level was investigated through QIIME-UCLUST by aligning the OTUs as a representative sequence against the metagenomic databases (Greengene). The phylum-level composition of microbes suggests that *Firmicutes* and *Bacteroidetes* are predominantly present in almost all samples, as shown in Figure 4. The overall presence of *Firmicutes*, *Actinobacteria*, *Bacteroidetes* are 75.3%, 14.4% and 7.8%, respectively. Comparative microbial analysis showed that the population of *Bacteroidetes* increased from the first to the 14th week, and in contrast, the population of *Firmicutes* decreased comparatively. The population composition of microbes in terms of class, order, family, genus and species was also shown in the Supplementary Figure S1A–D and Supplementary Table S4. Krona plots were also generated and provided into the Supplementary Figure S1E,F.

### 3.4. Phylogenetic Reconstruction

**Figure 5:**
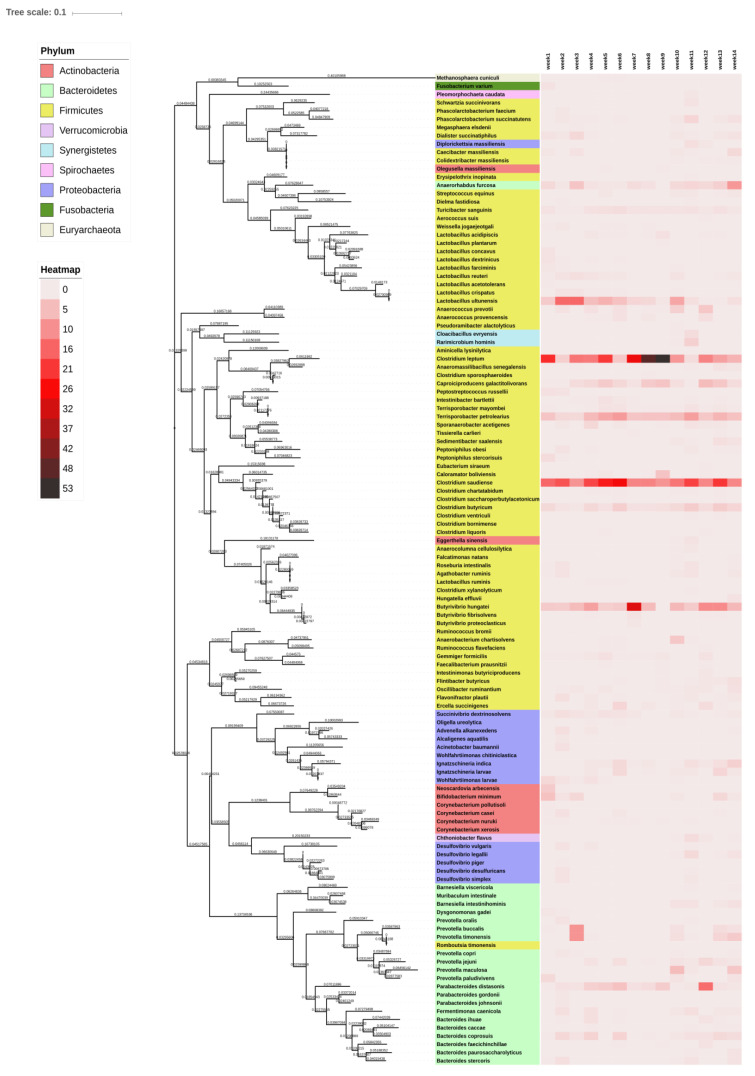
Phylum and species level phylogenetic tree of the 16S rRNA genes through maximum likelihood. Weekly sample indicated as column wise.

Phylogenetic heatmap result of the microbial species showing the differential enrichment at different weeks. As shown in Figure 5, the different phylums are indicated in different color and their respective species are clustered accordingly. The week-wise abundance of species is shown as the heatmap in color gradient coded (0–53). We found that some of the species, such as *Clostridium leptum*, *Clostridium saudience*, *Lactobacillus ultunensis*, *Terrisporobacter petrolearius*, and *Butyrivibrio hungatei* are significantly abundant, as shown in Figure 5. *Clostridium leptum* can be seen as highly abundant in weeks 7–9. We also performed jackknifed UPGMA clustering, as shown in supplementary Figure S2.

### 3.5. Linear Discriminant Analysis Effect Size Method

**Figure 6:**
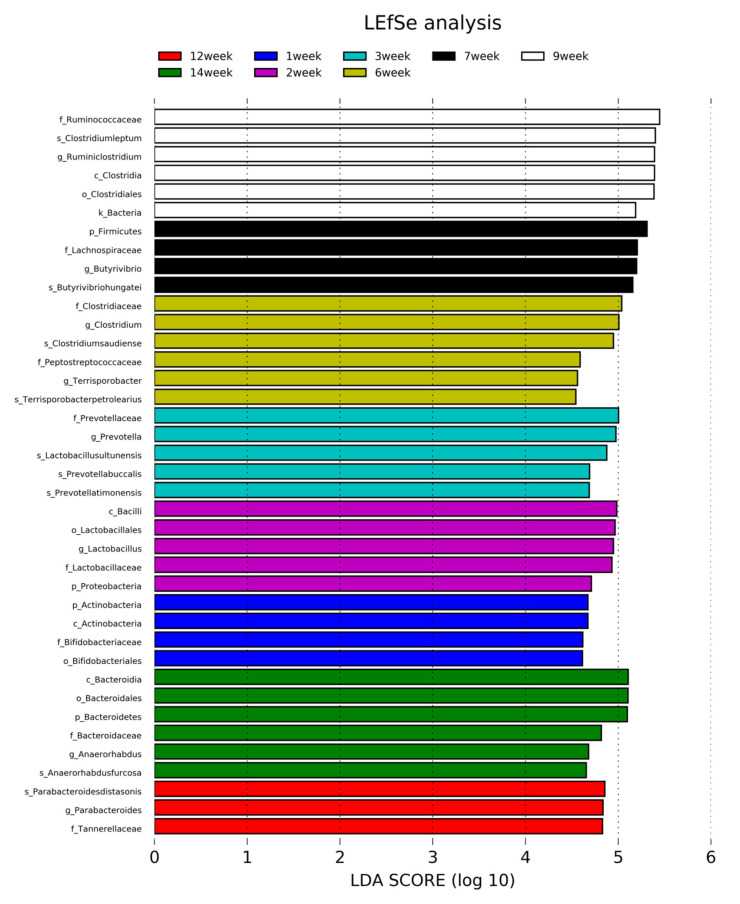
Marker microbiome identification through the linear discriminant analysis (LDA) effect size method (LEfSe), Color code is indicated according to week.

The species associated with different biological conditions can also be identified through linear discriminant analysis (LDA) effect size method (LEfSe). The LEfSe used random forest or iterative linear regression to identify the core as well as the unique microbiome. Through LEfSe, we identified the abundance of specific microbe in each week, as shown in Figure 6 with the weekly color-coded gradient indication. An LEfSe result also corroborates the findings of the phylogenetic analysis, as we observed *Rumino coccaceae*, *Clostridium leptum*, *Butyrivibrio hungatei*, *Clostridium saudiense*, *Lactobacillus ultunensis*, *Bacteroides stercoris*, *Desulfovibrio piger*, *Bifidobacterium minimum*, *Anaerorhabdus furcosa*, *Ignatzschineria larvae*, *Parabacteroides distasonis*, *Prevotella timonensis* are abundant, as shown in Figure 6.

### 3.6. Functional Annotations of Microbes

**Figure 7:**
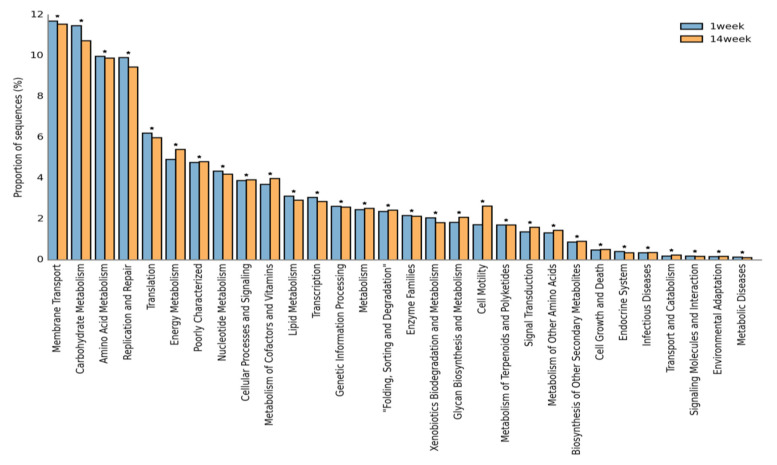
Functional annotation of the slurry bacterial population for the first and 14th weeks as the blue and yellow bars, respectively and * represents *p* ≤ 0.05.

On the basis of the KEGG pathway analysis, the functional behavior of the microbes presents in the slurry samples for the first and 14th weeks are shown in Figure 7. Some of the pathways, such as membrane transport, carbohydrate metabolism, amino acid metabolism, replication and repair, translation, and energy metabolism, are predominantly enriched. The percentage of OTUs vs. pathways has been plotted through the STAMP software (Figure 7).

### 3.7. Identification of Co-Occurring and Mutually Exclusive Microbes through Network Analysis

**Figure 8:**
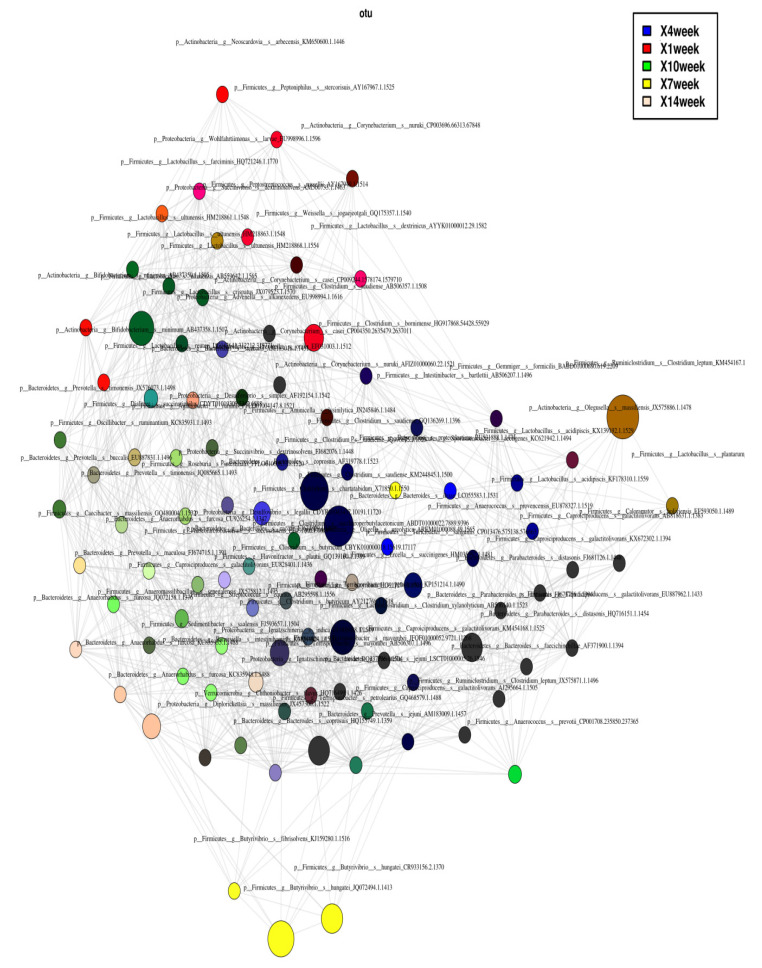
Operational taxonomic unit (OTU) co-occurrence network, where each node indicates the microbial OTU, and the size of the nodes indicates the number of degrees. Different colors indicate the weekly samples.

Through network analysis, we identified the abundance of particular taxa at a specific week of samples. By using Pearson’s correlation index method and highly significant (*p* < 0.05) correlation, highly correlated nodes (OTUs) were placed more closely and distant related nodes were located far from the hub region, as shown in Figure 8. The size and intensity of the respective color indicates the degree of abundance in the sample. We observed, at first week, that *Clostridium bornimense*, *Lactobacillus ultunensis*, and *Proteobacteria succinivibrio* were abundant; whereas, by the 14th week, *Diplorickettsia massiliensis* and *Anaerorhabdus furcosa* were abundant, as shown in Figure 8.

## 4. Discussion

The profiling of the bacterial content of swine slurry is important to understand their role in swine health and pig farming. Despite the early traditional method of metagenome analysis, NGS-based 16S rRNA sequencing technology enhances the capacity of biodiversity assessment from metagenome samples [35]. As 16S rRNA forms a part of bacterial ribosome and it contains regions of highly conserved and highly variable sequence. Sequencing costs are relatively cheaper as compared to shotgun sequencing so that we can amplify the only targets. In this study, an attempt was made to report the 16S marker changes into the swine slurry samples at different time points of storage. We observed that the pH of the slurry increased from 5.8 to 6.0 during the storage of slurry from the first to the 14th week. However, the moisture content of the slurry was increased to 97.3 by the 14th week. To avoid the pseudo replication during the sampling, we uniformly collected the sample from three different slurry pits to make three replicates of each time point.

We reported the taxonomic annotations of 42 samples starting from the first to 14th week of storage. Taxonomy at all levels, namely the phylum, class, family, genus, and the species of microbes present in the slurry samples, were examined at every week and provided into Supplementary Figure S1. We performed similarity-based OTU binning by directly comparing with the metagenomic database, by keeping a cutoff value of 97% in the species level. A rarefaction curve was used to measure the richness of the observed OTUs of the slurry samples against the reference OTUs [36]. Our richness curve results plotted through the Chao1, Observed species, Shannon, and Simpson indexes showed that the evenness of diversity was achieved in almost all samples. Beta-diversity through the Weighted UniFrac PCoA plot showed that the clusters of bacterial populations according to the early, mid, and late week.

Our study indicates that *Firmicutes* was the most abundant phylum present in almost all samples. However, the population of *Firmicutes* decreased from the first to the 14th week. In contrast, the population of *Bacteroidetes* increased from the first to the 14th week. Similar results were reported by the Shaufi, Mohd Asrore Mohd, et al. (2015) in the case of chicken gut microbial population [37]. It is well known and extensively reported that the microbiota present around the pig stable affect the health of the pig and subsequently affect the production of pig [38]. It is also documented that the pig fecal material is the primary source of airborne bacteria, such as *Clostridium leptum*, and *Clostridium saudiense*. However, *Clostridium leptum* is also known as carbohydrate-fermenting bacteria [39]. It is dominantly present in human fecal microbiota, at around 16–25% and the alteration of its composition may act as an indicator of many diseases [40]. Furthermore, *Clostridium saudiense,* a Gram-positive bacteria, was isolated from the human fecal sample. It is a dominant species present (9–18%) in almost all livestock gut microbiome. *Clostridium* species are known for helping the host organism against the pathogenic bacteria. It also helps in the digestion process, and methane production [41]. However, the specific function of *Clostridium saudiense* is still needs to be explored in swine gut microbiome [42].

Furthermore, *Butyrivibrio hungatei* is also abundant in almost all samples; it is a Gram-negative, and butyrate-producing bacteria [43]. *Butyrivibrio* species are known for helping the host by breaking down the protein, degrading the fiber, lipid biohydrogenation, etc. [44]. These are also reported as the producers of microbial inhibitors, as well as to help in the degradation of plant carbohydrate hemicellulose [45]. *Lactobacillus ultunensis* is a *Lactobacillus* species isolated from the human gastric environment [46]. *Lactobacillus* is a Gram-positive, anaerobic, and lactic acid-producing bacteria, abundantly present in healthy human, livestock and other adult organisms [47]. It is administered to infants in adequate amounts as a probiotics to improve the host health [48]. *Lactobacillus* species are reported as helpful for combating against several diseases, such as inflammatory bowel disease, type 1 diabetes, celiac disease and multiple sclerosis [49].

LEfSe is a tool for the discovery of biomarker bacteria among various samples by analyzing the abundance of microbes [50]. We also implemented this tool to analyze the microbial biomarkers in our swine slurry data, and found that *Clostridium* and *Lactobacillus* were predominant across the samples and corroborated the earlier findings [51]. We reported the specific bacterial biomarkers in each week of storage period. The overall bacterial abundance and their diversity may reflect a similar association with the pig GIT ecosystem. The characterization of the functional pattern of the microbiome is essential for the comparative metagenomic analyses of pig slurry.

The relevant metabolic activities of microbes are typically species or strain specific. We are aware that the prediction of the functional activities of microorganisms on the basis of the V4 region of 16S rRNA genes may not provide exact or relevant information regarding their functional pattern. The pathway analysis to identify enriched functions of the microbes present, as determined by 16S RNA analysis may add little to the results of the study. However, we assumed that it may give the preliminary idea about the inference of biological functions of the microbes, and a lot may be done before an exact conclusion.

We predicted the KEGG pathway based on the retrieval of homologous genes into the sample. We observed little differences at the functional level because of their storage time. The KEGG pathways, such as membrane transport, carbohydrate, and amino acid metabolism were significantly enriched in across the sample. There is evidence that the microbes present in the sample such as fecal matter, cecum content, sludge, slurry, and GIT microbes are greatly involved in multiple pathways of the host organism [52,53]. Cai, Mingwei, et al. [54] reported that metagenomic sequences of municipal sludge are involved in carbohydrate and protein metabolism during wastewater biogas-producing systems. As shown in Figure 7, carbohydrate metabolism, replication and repair, cell motility, etc. have shown some differences between the two groups. Since we provided the same food throughout the experimental duration to all the pigs, we did not expect the functional variations to be because of feedings.

The co-occurring and mutually exclusive network showed that *Clostridium bornimense, Lactobacillus ultunensis*, *Proteobacteria succinivibrio* were abundant in first week. By the 14th week *Diplorickettsia massiliensis*, *Anaerorhabdus furcosa* were abundant. This was reported that *Clostridium bornimense* was involved in essential metabolic pathways in the biogas fermentation process. Many species of *Clostridium* genus were involved in methanogenesis, which may indicate that the production of methane starts at very early stages of slurry storage. *Anaerorhabdus furcosa*, anaerobic bacteria, produces ethanol by breaking the pentose from the sewage sludge. This also indicates that the production of ethanol starts in the latter stage of slurry storage.

## 5. Conclusions

As pig production is significant in terms of agriculture and livestock industry, the management of their slurry waste is needed. Our study represents the longitudinal metagenomic profiling of swine slurry to understand the bacterial community during its storage in the slurry pit. Weekly comparison among samples clearly indicates that there were significant changes in the dominant microbial community during the first to 14th week. The majority of bacterial populations belonged to *Firmicutes* and *Bacteroidetes*. However, the abundance of *Firmicutes* decreased from the first to the 14th week, whereas the abundance of *Bacteroidetes* increased from the first to the 14th week. It was reported that the strains of such bacteria were used as microbial markers and their relative abundance may be governed by fermentation conditions. We found that some of the microbial species, such as *Clostridium leptum*, *Clostridium saudiense*, *Butyrivibrio hungatei*, *Lactobacillus ultunensis*, are abundantly present across the samples. Furthermore, the pathogenic indicator, such as *Escherichia fergusonii,* which was reported as linked with pneumonia in cattle, was also present in the slurry samples. We also reported functional annotations of these microbes and found their involvement in many pathways such as membrane transport, carbohydrate and amino acid metabolism, replication and repair, and energy metabolism. Overall, our findings improve the understanding of microbial abundance in the swine slurry in a time series manner.

## Supplementary Materials

The following are available online at https://www.mdpi.com/2076-2615/10/8/1372/s1, Figure S1: Taxonomic annotation from the first to 14th weeks (**A**) class, (**B**) group, (**C**) order, (**D**) species, (**E**) krona chart of the first week, (**F**) krona chart of the 14th week. Figure S2: Jackknifed unweighted pair group method arithmetic mean (UPGMA) clustering phylogenetic map. Figure S3: Rarefaction curves. Table S1: Statistics of raw data. Table S2: Alpha diversity, details of number of OTUs, Chao1, Shannon, Simpson, and Goods Coverage. Table S3: OTU identifier table. Table S4: Taxonomy details.
