# Supplementary figures and images for: Compositional and Functional Characteristics of Swine Slurry Microbes through 16S rRNA Metagenomic Sequencing Approach
Source: Animals (Basel). 2020 Aug 7;10(8):1372. doi: 10.3390/ani10081372 (PMC7460454; doi:10.3390/ani10081372)

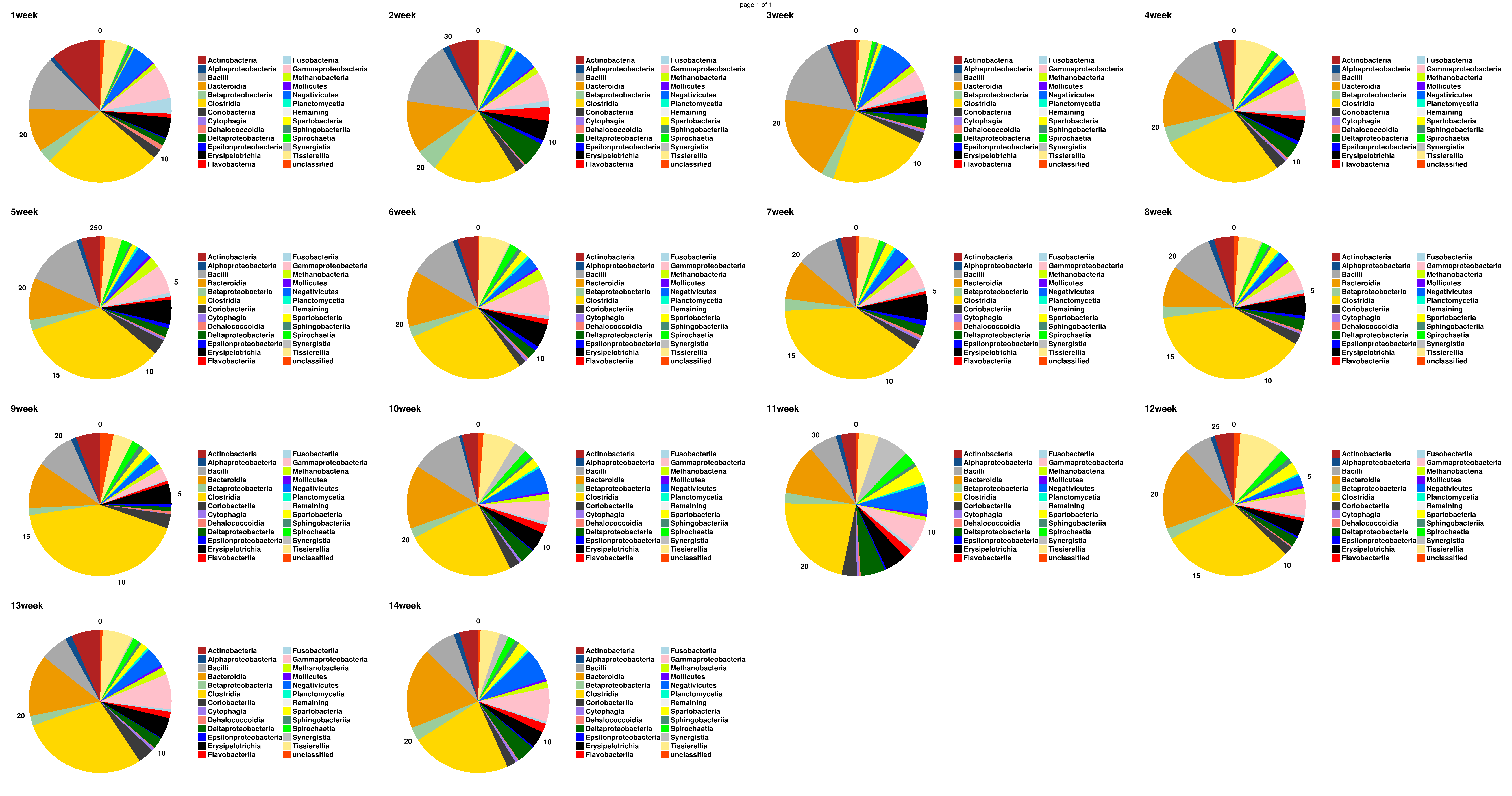

Supplement: Supplementary file 1 [file animals-10-01372-s001.zip › animals-871872-supplementary/Figure_S1A_pie_class_group.png]

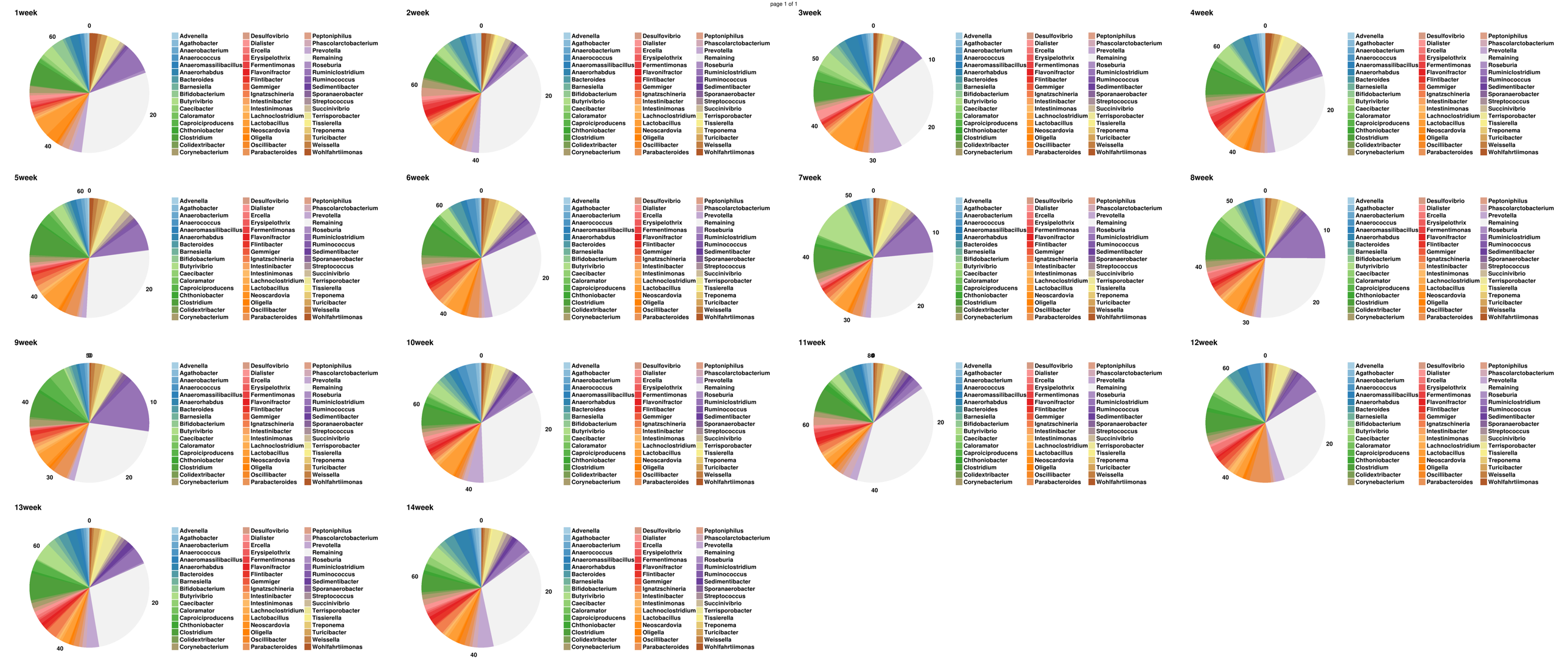

Supplement: Supplementary file 1 [file animals-10-01372-s001.zip › animals-871872-supplementary/Figure_S1B_pie_g_group.png]

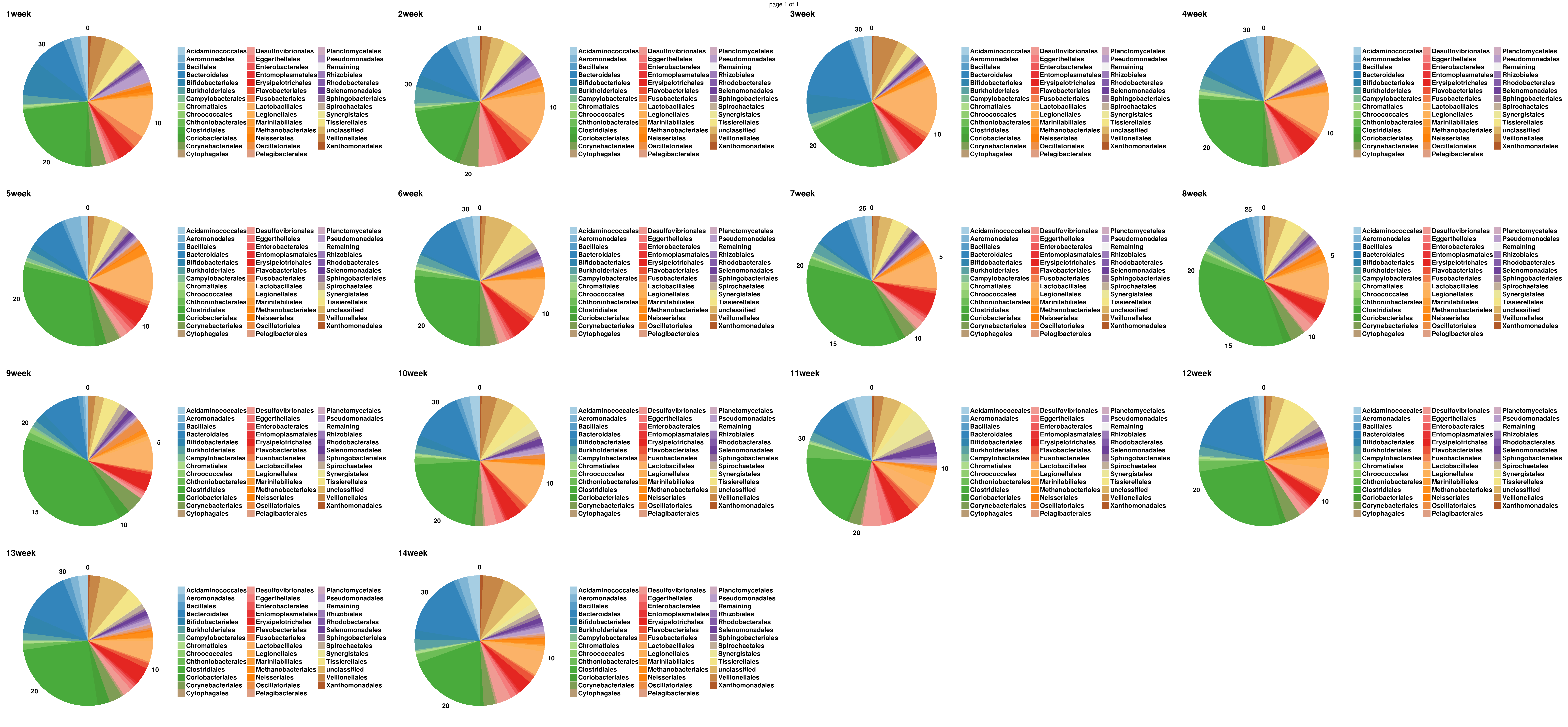

Supplement: Supplementary file 1 [file animals-10-01372-s001.zip › animals-871872-supplementary/Figure_S1C_pie_o_group.png]

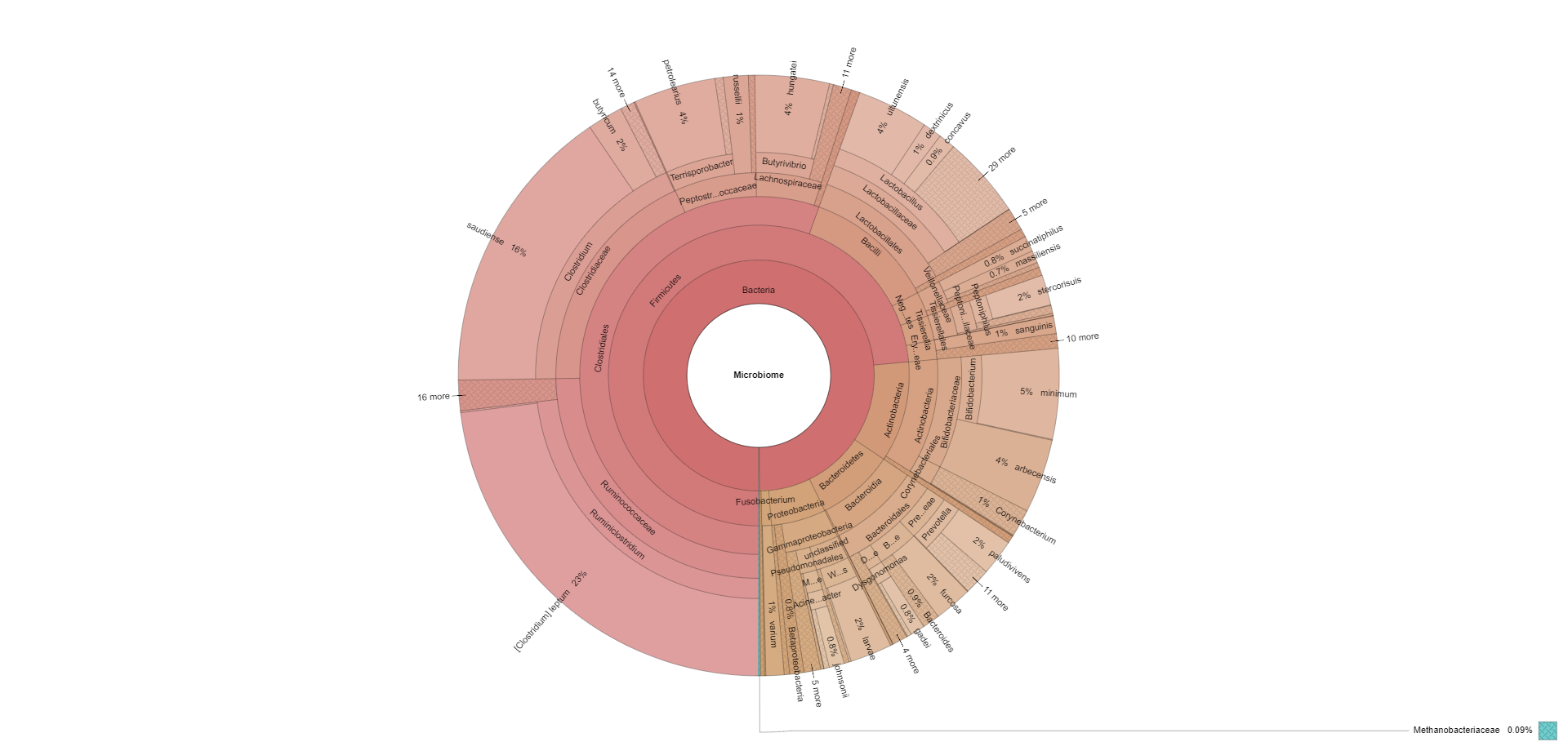

Supplement: Supplementary file 1 [file animals-10-01372-s001.zip › animals-871872-supplementary/Figure_S1E_krona1week.png]

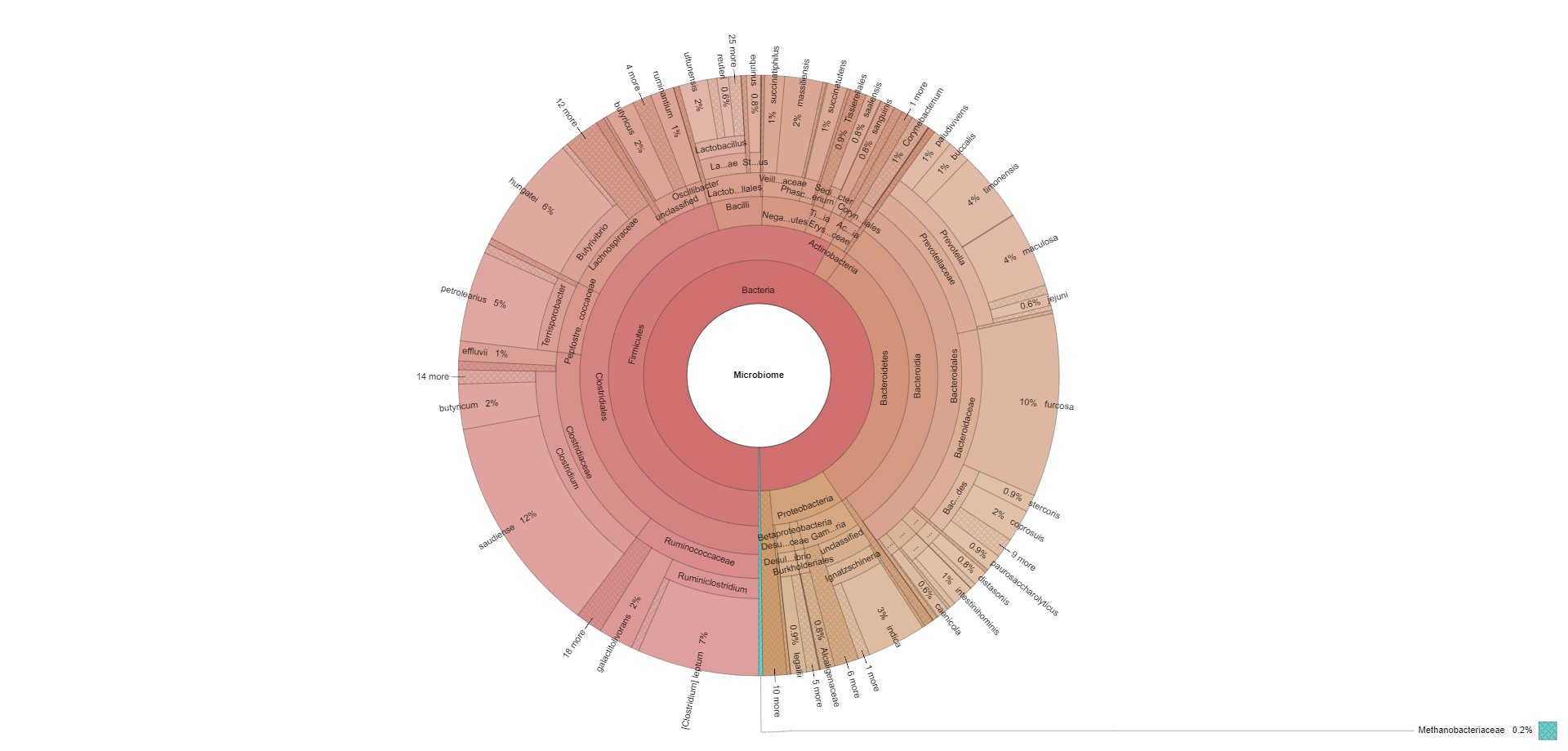

Supplement: Supplementary file 1 [file animals-10-01372-s001.zip › animals-871872-supplementary/Figure_S1F__krona14week.png]

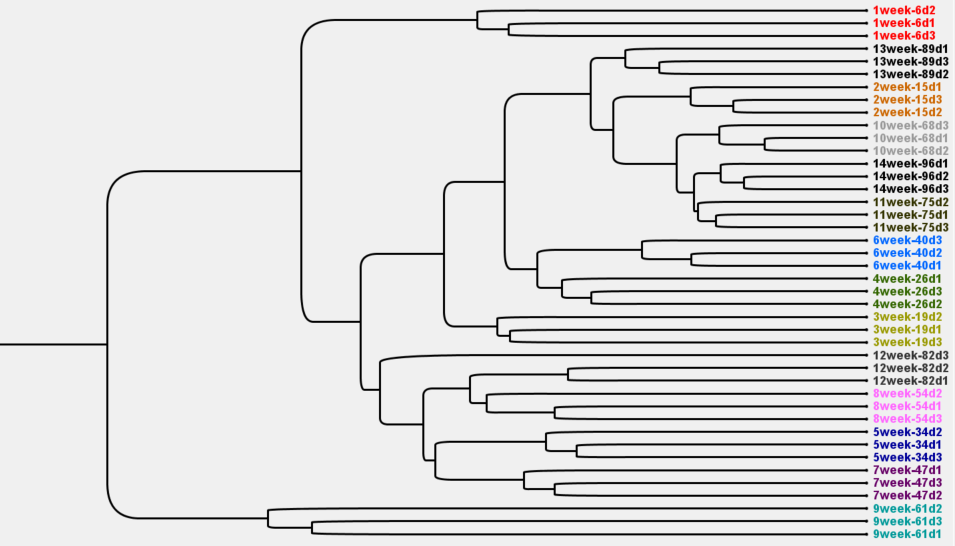

Supplement: Supplementary file 1 [file animals-10-01372-s001.zip › animals-871872-supplementary/Figure_S2.png]

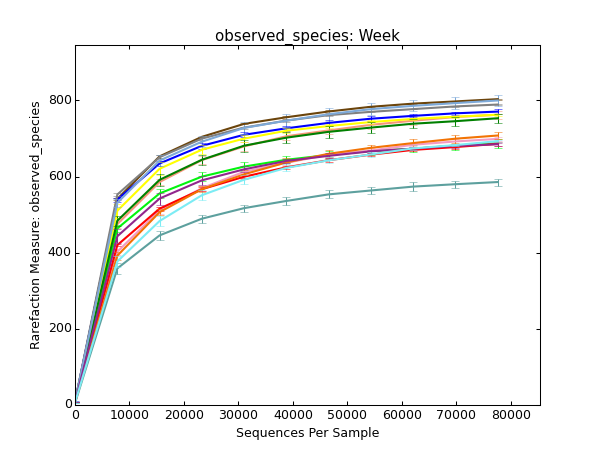

Supplement: Supplementary file 1 [file animals-10-01372-s001.zip › animals-871872-supplementary/Figure_S3_A_observed_speciesWeek.png]

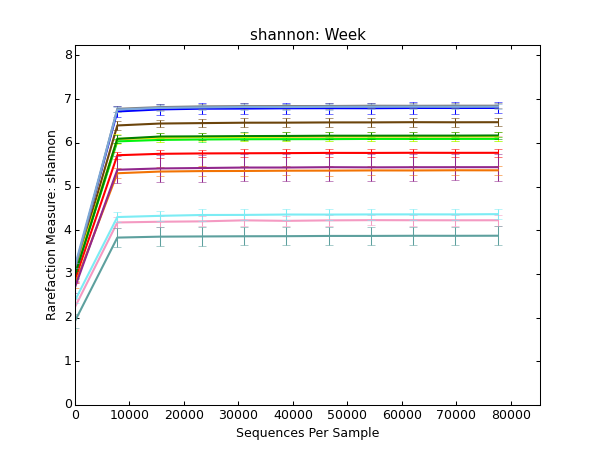

Supplement: Supplementary file 1 [file animals-10-01372-s001.zip › animals-871872-supplementary/Figure_S3_B_shannonWeek.png]

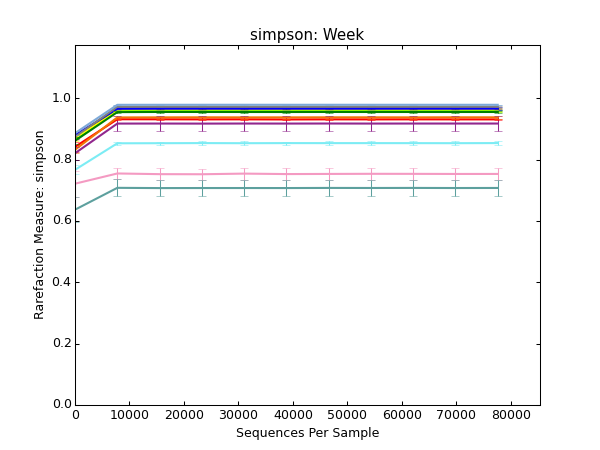

Supplement: Supplementary file 1 [file animals-10-01372-s001.zip › animals-871872-supplementary/Figure_S3_C_simpsonWeek.png]
